# Metaproteomics characterizes human gut microbiome function in colorectal cancer

**DOI:** 10.1038/s41522-020-0123-4

**Published:** 2020-03-24

**Authors:** Shuping Long, Yi Yang, Chengpin Shen, Yiwen Wang, Anmei Deng, Qin Qin, Liang Qiao

**Affiliations:** 1 Department of Chemistry, Shanghai Stomatological Hospital, Fudan University, Shanghai, China; 2 Changhai Hospital, The Naval Military Medical University, Shanghai, China; 3 Department of Clinical Laboratory Medicine, Shanghai Tenth People’s Hospital of Tongji University, Shanghai, China; 4 Shanghai Omicsolution Co., Ltd., Shanghai, China

**Keywords:** Microbiome, Bacteria

## Abstract

Pathogenesis of colorectal cancer (CRC) is associated with alterations in gut microbiome. Previous studies have focused on the changes of taxonomic abundances by metagenomics. Variations of the function of intestinal bacteria in CRC patients compared to healthy crowds remain largely unknown. Here we collected fecal samples from CRC patients and healthy volunteers and characterized their microbiome using quantitative metaproteomic method. We have identified and quantified 91,902 peptides, 30,062 gut microbial protein groups, and 195 genera of microbes. Among the proteins, 341 were found significantly different in abundance between the CRC patients and the healthy volunteers. Microbial proteins related to iron intake/transport; oxidative stress; and DNA replication, recombination, and repair were significantly alternated in abundance as a result of high local concentration of iron and high oxidative stress in the large intestine of CRC patients. Our study shows that metaproteomics can provide functional information on intestinal microflora that is of great value for pathogenesis research, and can help guide clinical diagnosis in the future.

## Introduction

Colorectal cancer (CRC) is the third most commonly diagnosed cancer and the fourth leading cause of oncological mortality worldwide^1^. In recent years, CRC incidence rates in developing countries have been rising because of obesity and westernized diet^2^. In particular, high-level intake of red meat and inadequate intake of vegetables and fiber could increase the risk of CRC^2^. The pathogenesis of CRC is a complex multistep process involving genetic alterations^3^, immune factors^4^, environmental factors (e.g. diet and lifestyle)^5^, and human gut microbiome^6^.

Human gut hosts about 100 trillion microbes. Most microbes colonize the large intestine at a concentration of about 10^12^ cell per mL^7^. Emerging evidences indicate that microbial dysbiosis is a driving force in the pathogenesis of intestinal tumor^8^. Studies using metagenomics-based approaches demonstrated that *Parvimonas micra*, *Solobacterium moorei*, *Fusobacterium nucleatum*, and *Peptostreptococcus stomatis* are enriched in the gut of CRC patients^9^. It has been observed that the enterotoxigenic *Bacteroides fragilis* is increased in the feces and colonic mucosa of CRC patients^10,11^. Tjalsma et al. presented a bacterial driver–passenger model for CRC pathogenesis, indicating that CRC can be initiated by “driver” bacteria that are eventually replaced by “passenger” bacteria during tumorigenesis^6^. However, the actual function of human gut microbiome in the pathogenesis of CRC remains largely unexplored. There is an urgent need to fully understand the impact of microbes in CRC.

Traditional methods for bacterial characterization are usually based on bacterial culture. Culturomics is a bacterial identification method that combines multiple culture strategies, matrix-assisted laser desorption/ionization–time of flight mass spectrometry (MS) identification, and 16S rRNA typing^12^. However, most microbes in the gut are difficult to culture. Metagenomics has recently been widely used to characterize gut microbiome without bacterial culture^13^. The methods can provide information on the taxonomic abundances of samples. Nevertheless, biases can exist due to DNA extraction methods, the use of amplification primers, and bioinformatic tools^12,13^. In addition, sequencing cannot distinguish between live bacteria and transient DNA^12^. It is also difficult to reveal important functional elements of gut microbiome solely by metagenomics. Therefore, it has been suggested that functional omics, like metaproteomics and metabolomics, should also be involved in the study of gut microbiome, wherein “function first, taxa second” has been proposed^14^.

Metaproteomics was initially used to study the microbial function of environmental samples, like soil, activated sludge, and acid mine drainage^15^. In 2009, Verberkmoes et al. first studied human gut microbiome using shotgun metaproteomics, wherein the samples were feces collected from a pair of monozygotic twins^16^. In 2017, Tanca et al. chose a cohort of 15 healthy Sardinian populations and studied the function of their gut microbiome using metaproteomics^17^. In 2018, Zhang et al. demonstrated an upregulated expression of human proteins related to oxidative antimicrobial activity in pediatric inflammatory bowel disease (IBD) by metaproteomics^18^.

Herein, we used data-independent acquisition (DIA)-based label-free quantitative proteomics for a cohort analysis of CRC patients’ and healthy volunteers’ gut microbiome. A total of 30,062 protein groups and 91,902 peptides from 195 genera of microbes were identified and quantified. Three hundred and forty-one protein groups were found significantly different in abundance between the CRC patients and the healthy volunteers. Among the 341 proteins, 27 are related to iron intake and transport, and 42 are related to oxidative stress, which can be resulted from the high local concentration of iron and high oxidative stress in the large intestine of CRC patients. The results show that not only taxonomic abundances but also function of gut microbiome is changed during the pathogenesis of CRC.

## Results

### Metaproteomic characterization of the gut microbiome of CRC patients and healthy crowds

**Fig. 1:**
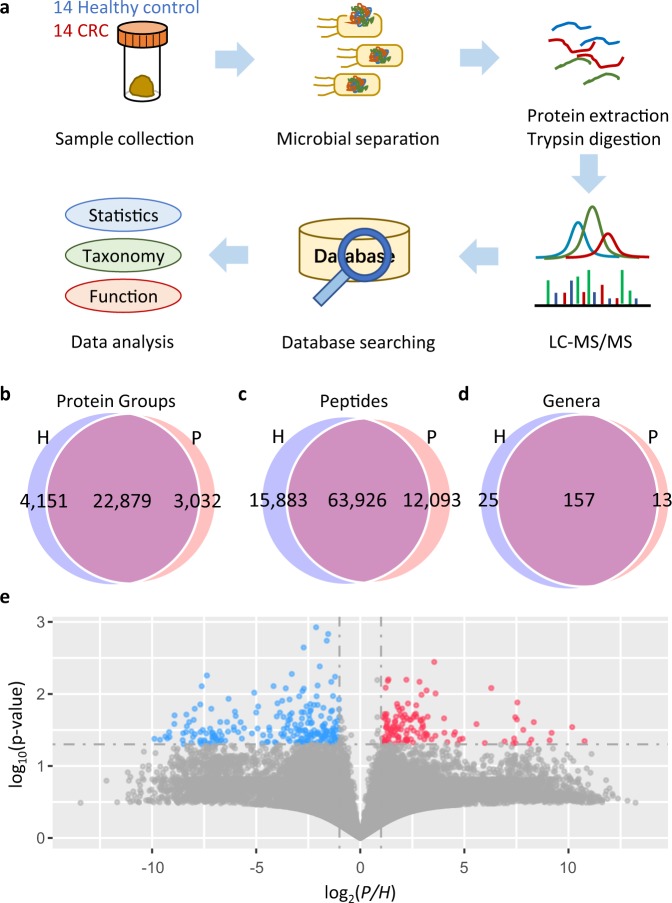
Metaproteomic characterization of the gut microbiome of CRC patients and healthy crowds. **a** Experimental design and workflow. The numbers of protein groups (**b**), peptides (**c**), and genera (**d**) identified from the CRC patient group (P) and the healthy volunteer group (H). **e** Volcano plot indicating the differential proteins. Protein groups with P/H fold change (FC(P/H)) ≥ 2 and *P* value < 0.05 were colored red, while those with FC(P/H) ≤ 0.5 and *P* value < 0.05 were colored blue. Source data are provided as a Source Data file.

In the study, we enrolled 14 CRC patients and 14 healthy volunteers. There was no significant difference in ages or body weights between the two groups (Supplementary Table 1). As illustrated in Fig. 1a, gut microbes were enriched using differential centrifugation. After protein extraction and trypsin digestion, label-free DIA was used to identify and quantify proteins in each sample using a merged spectral library generated by data-dependent acquisition (DDA) experiments performed on a pool from every sample and spectrum-centric analysis of the DIA data. The workflow of library generation is described in detail in the “Methods” section and Supplementary Fig. 1. De novo sequencing-assisted database searching by PEAKS^19^ was conducted on the pooled fractionated DDA data against successively a database of stool from Human Microbiome Project (HMP) (containing >4.8 million protein entries) and a database combining the National Center for Biotechnology Information (NCBI) non-redundant (nr) bacteria (containing >78 million protein entries) and the SwissProt human (>20,000 protein entries). At the protein group level, 15,685 protein groups were identified, including 11,391 (72.6%) from the HMP database and 3920 (25.0%) from the NCBI nr database (Supplementary Fig. 1a and Supplementary Data 1). Most of the identified protein groups are from microbes, indicating a good sample pretreatment by the differential centrifugation. Then the DDA and DIA data were searched against a database combined from the HMP and the identified NCBI nr proteins (11,994 entries) by SpectroMine, and the results are shown in Supplementary Fig. 1b and Supplementary Data 2. Consequently, 36,053 protein groups and 103,444 peptides were identified from the pooled DDA data with an identification rate of MS/MS spectra of 26.7% (178,300 peptide-spectrum matches (PSMs) from 668,162 MS/MS spectra). From the DIA data, 12,463 protein groups and 39,319 peptides were identified with an identification rate of 33.3% (794,028 PSMs from 2,386,773 MS/MS spectra) by spectrum-centric database searching. The search results were merged to generate spectral library for peptide-centric DIA analysis, and finally the library contained 37,416 protein groups and 112,436 peptides corresponding to 210 genera of microbes (Supplementary Fig. 1c).

From the 28 samples, 30,062 protein groups and 91,902 peptides were identified and quantified by peptide-centric DIA analysis (Fig. 1b, c, and Supplementary Data 3). Taxonomic information was assigned to 78,391 peptides. Among them, 36,244 peptides were matched to 114 families of microbes and 33,690 peptides to 195 genera. One hundred and fifty-seven genera were shared between the healthy control group and the CRC patient group, while 25 were found only in the healthy crowds and 13 only in the CRC patients (Fig. 1d, and Supplementary Data 4). In average, 21,510 ± 5760 (mean ± standard deviation, sic passim) peptides and 9078 ± 2225 protein groups were identified per sample from the healthy volunteers group, and 18,368 ± 6941 unique peptides and 7761 ± 2663 protein groups were identified per sample from the CRC patient group. It is worth noting that 17% more proteins were identified from the healthy crowds than the CRC patients, indicating a lower diversity of microbial proteins in the CRC patients. During the proteomic experiments, independent retention time (iRT) peptides were added to calibrate RT. No obvious changes in the MS1 and MS2 intensities of the iRT peptides were observed (Supplementary Fig. 2) during the 28 DIA runs, demonstrating that there was not an obvious change of MS sensitivity during the experiments. The median full width at half maximum of chromatographic peaks in each sample was 0.294 ± 0.017 min for the healthy control group and 0.291 ± 0.024 min for the CRC patient group. The numbers of data points per peak were 9.1 ± 0.6 and 8.8 ± 0.9 for the healthy control and CRC patient groups, respectively.

Owing to the complexity of fecal sample, heterogeneity among samples, and the large numbers of identified proteins, no significant difference in protein abundance between the two groups could be reached with a significance cut-off of a Benjamini–Hochberg adjusted *P* value < 0.05. The same situation has been reported in previous metaproteomic research^20,21^. Therefore, differentially expressed proteins were determined using fold change (FC) analysis and *t* test. Proteins with FC ≥ 2 and *P* value < 0.05 were accepted as differentially expressed proteins. Three hundred and forty-one differential proteins between the CRC patients (P) and healthy crowds (H) were discovered, shown in the volcano plot (Fig. 1e) and Supplementary Data 5.

### Taxonomy of the gut microbiome of CRC patients and healthy crowds based on quantitative metaproteomics

**Fig. 2:**
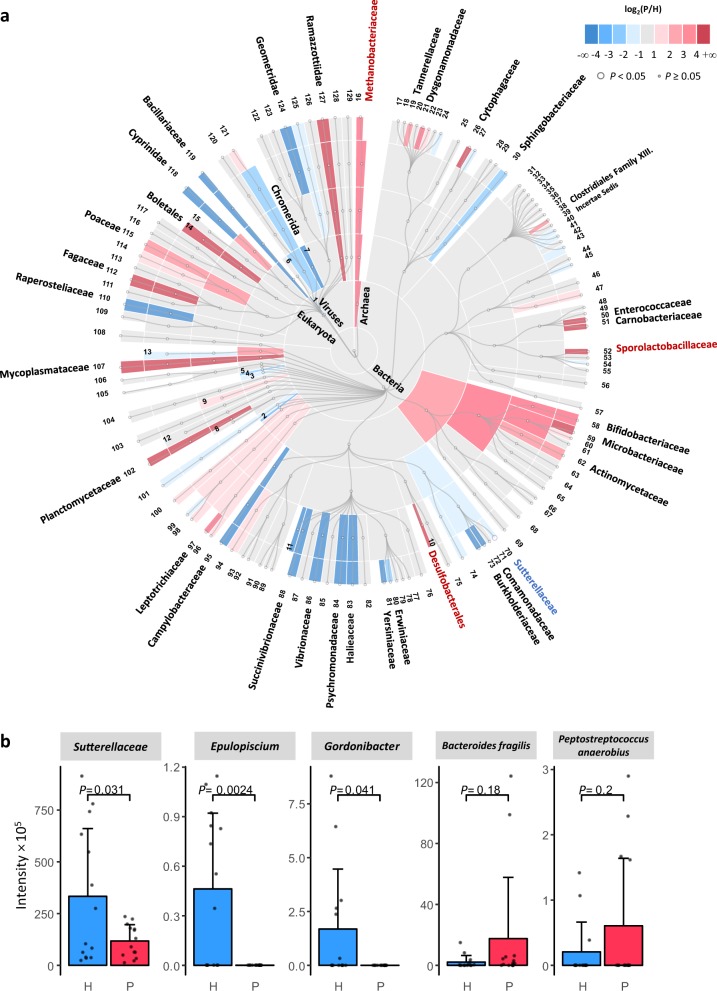
Taxonomic abundances of the gut microbiome of the CRC patients (P) and healthy crowds (H). **a** Cladogram illustrating taxa (domain to family) abundances in the two groups. Colors indicate the fold change log_2_(P/H). Large circles indicate *P* value < 0.05. Information on the phylum, class, order, and family of the labeled numbers is given in Supplementary Data 6. **b** Bar charts showing the selected differential families, genera, and species between the CRC patients and the healthy controls. The bars show the average of 14 samples in each group. Error bars indicate standard deviation. Individual data points are overlaid as dots. Taxa are selected for presentation in **a** and/or **b** if significant differences of their abundance between CRC patients and healthy people have been reported in previous studies or are observed (*P* value < 0.05) in this work. The selected classes and families are highlighted in **a** with red or blue color of the taxa name. Source data are provided as a Source Data file.

The density of bacteria in large intestine is very high (10^12^ cells per mL), and some researchers have suggested that CRC could be a bacteria-related disease^22^. Herein, we utilized the quantitative information of all the identified peptides to display the taxonomic abundances of the gut microbiome of the CRC patients compared to that of the healthy controls, Fig. 2a and Supplementary Data 6. We observed some abundance differences in taxa that have been reported in previous metagenomic studies^8,23^. At the order and family level, Desulfobacterales, *Methanobacteriaceae*, and *Sporolactobacillaceae* showed higher abundance in the CRC patients than in the healthy crowds (Fig. 2a). Desulfobacterales are sulfate-reducing bacteria that can oxidize lactate, pyruvate, and molecular hydrogen during the reduction of sulfate to generate hydrogen sulfate that is toxic to intestinal epithelium cells^24^. *Methanobacteriaceae* are strict anaerobes, which can produce methane by reducing carbon dioxide and molecular hydrogen^25^. Some studies have suggested that excessive methane production could lead to CRC^26,27^. *Sporolactobacillaceae* and *Methanobacteriaceae* compete for molecular hydrogen utilization. It has been reported that interaction between *Sporolactobacillaceae* and *Methanobacteriaceae* in the large intestine can contribute to the pathogenesis of CRC^27^. At the species level, we found that *B. fragilis* and *Peptostreptococcus anaerobius* were more abundant in the CRC group, (Fig. 2b), which is consistent with previously reported metagenomic results^8,26^.

Based on the metaproteomic data, we have also observed significant changes (*P* value < 0.05) in taxonomic abundance that have not been revealed by metagenomics. Indeed, it has been reported that taxonomic abundances based on metagenomics and metaproteomics are different^17^. As shown in Fig. 2a, b, the family *Sutterellaceae* was more abundant in the healthy crowds (*P* value = 0.031, FC(P/H) = 0.341). At the genus level, *Epulopiscium* (*P* value = 0.002, FC(P/H) < 0.001) and *Gordonibacter* (*P* value = 0.041, FC(P/H) < 0.001) were more abundant in the healthy crowds.

### Differential gut microbial proteins between CRC patients and healthy controls

**Fig. 3:**
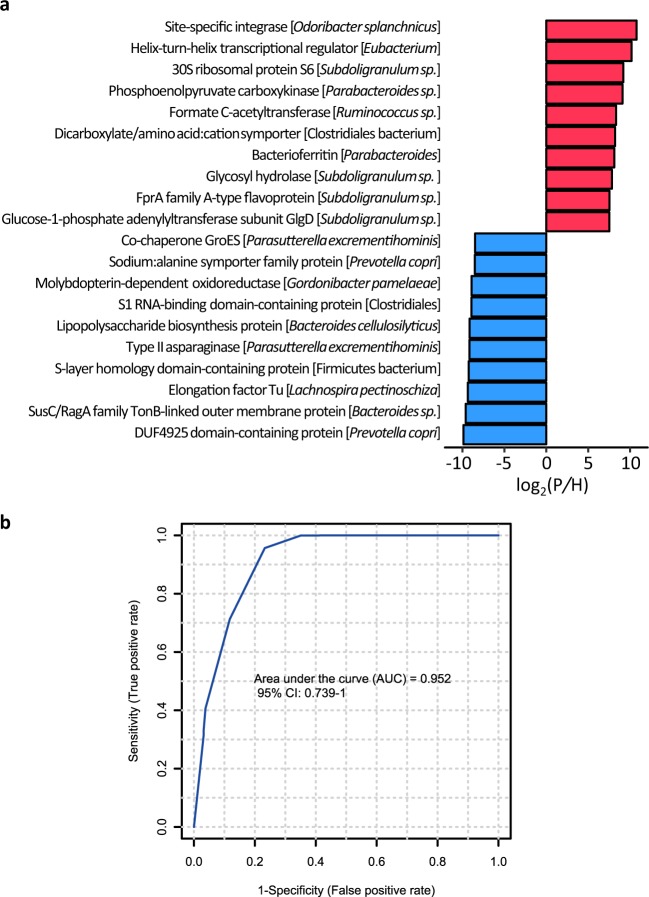
Top 20 differential proteins between the CRC patients and the healthy controls. **a** The 10 proteins with the largest fold change (FC(P/H)) (red) and the 10 proteins with the smallest FC(P/H) (blue). **b** Receiver operating characteristic (ROC) curve using the 20 proteins as potential candidate biomarkers for CRC diagnosis. Linear support vector machine (LSVM) was used as a classification method, and 100 rounds of Monte-Carlo cross-validation were performed to generate the ROC curve. Details are described in the “Methods” section. Source data are provided as a Source Data file.

Among the 341 differential proteins, 124 were more abundant in the CRC patients, and 217 were more abundant in the healthy controls. The 10 proteins with the largest FC(P/H) were from the genera of *Odoribacter*, *Eubacterium*, *Subdoligranulum*, *Parabacteroides*, and *Ruminococcus*, as well as the Clostridiales order, Fig. 3a. Site-specific integrase, the protein with the largest FC(P/H) from *Odoribacter*, involves in DNA binding, DNA integration, and DNA recombination. Bacterioferritin from *Parabacteroides*, the protein with the seventh largest FC(P/H), is an iron storage protein that participates in iron ion transportation and cellular iron ion homeostasis. The 10 proteins with the smallest FC(P/H) were from *Prevotella*, *Bacteroides*, *Lachnospira*, *Firmicutes*, *Parasutterella*, *Gordonibacter*, and Clostridiales, Fig. 3a. The proteins are related to protein folding, transmembrane transport, asparagine metabolism, RNA binding, or lipopolysaccharide synthesis. We assessed whether the 20 most differential proteins could be used as potential candidate biomarkers in clinical CRC diagnosis. Linear support vector machine (LSVM) was used as a classification method. Cross-validations (described in the “Methods” section) were performed to generate the receiver operating characteristic (ROC) curve as shown in Fig. 3b, where the area under the curve was 0.952, indicating that the 20 gut microbial proteins are potential candidate biomarkers for CRC diagnosis.

### Functional characteristics of the intestinal microbiome of CRC patients

**Fig. 4:**
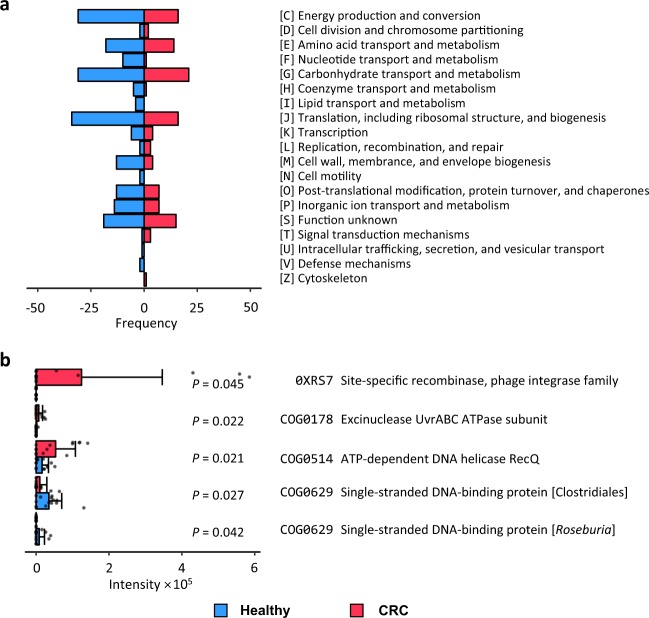
Clusters of orthologous groups (COG) categories of the 341 differential proteins (*P* value < 0.05). **a** Numbers of proteins in each COG category; red: more abundant in CRC patients, blue: more abundant in healthy crowds. **b** Bar plots of differential proteins in COG category L (DNA replication, recombination and repair); red: gut microbial proteins in CRC patients, blue: gut microbial proteins in healthy crowds. The bars show the average of 14 samples in each group. Error bars indicate standard deviation. Individual data points are overlaid as dots. Source data are provided as a Source Data file.

We annotated functions of the differential proteins using eggNOG^28^. The 341 differential proteins were annotated in 19 clusters of orthologous groups (COG) categories (shown in Fig. 4a). For most of the COG categories, more proteins were found with high relative abundance in the healthy control group than in CRC patient group. However, we found that there were more gut microbial proteins related to DNA replication, recombination, and repair (category L) that were more abundant in the CRC patient group compared to the healthy control group. Excinuclease UvrABC ATPase subunit (*P* value = 0.022, FC(P/H) = 16.3) and ATP-dependent DNA helicase RecQ (*P* value = 0.021, FC(P/H) = 3.29) related to DNA repair and SOS response were more abundant in the gut microbes of the CRC patients than the healthy crowds, Fig. 4b. The observation is in accordance with a previous report that the functions of microbial proteins related to DNA replication, recombination, and repair are among the most significantly increased functions of gut microbes in the patients of IBD^18^.

### Gut microbial proteins in CRC patients related to iron intake/transport and oxidative stresses

**Fig. 5:**
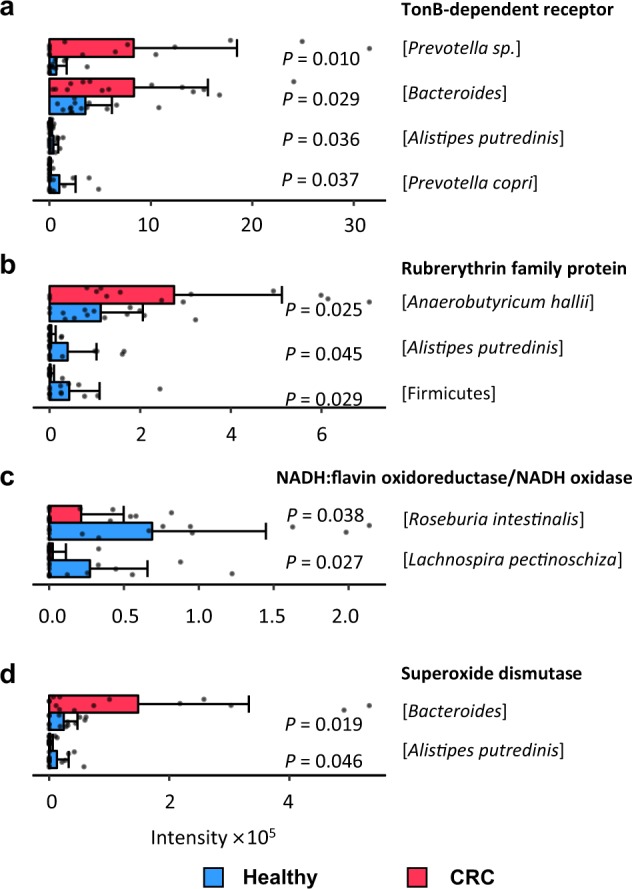
Quantitative comparison of proteins related to iron intake and transport and oxidative stress between the CRC patient group (red) and the healthy control group (blue). **a** TonB-dependent receptors, **b** rubrerythrin family proteins, **c** nicotinamide adenine dinucleotide (NADH):flavin oxidoreductase/NADH oxidases, **d** superoxide dismutases (SODs). The bars show the average of 14 samples in each group. Error bars indicate standard deviation. Individual data points are overlaid as dots. Source data are provided as a Source Data file.

It has been reported that the increased risk of CRC caused by red meat intake is mainly due to a high amount of heme iron in red meat^29^. There are evidences showing that excessive iron is associated with the development and progression of CRC^30,31^. Iron was found accumulated in tumors of CRC patients, and studies have shown that elimination of free iron by chelation can inhibit the growth of CRC cells^29,32^. By gene ontology (GO) annotation^33^, we found that 27 of the 341 differential proteins are related to iron intake and transport (Supplementary Table 2). TonB-dependent receptors can regulate iron concentration in human intestinal lumen^34^. In our results, TonB-dependent receptors from *Prevotella* sp. (FC(P/H) = 12.2, *P* value = 0.010) and *Bacteroides* (FC(P/H) = 2.35, *P* value = 0.029) were more abundant in the CRC patients compared to the healthy crowds, Fig. 5a, which could be a result of the high iron concentration in the large intestine of the CRC patients. Rubrerythrin is di-iron protein that belongs to the ferritin-like superfamily and is involved in iron storage, iron detoxification, and oxidative stress response^35^. Our results show that rubrerythrin family protein from *Anaerobutyricum hallii* was more abundant in the CRC patients compared to the healthy crowds (FC(P/H) = 2.44, *P* value = 0.025), Fig. 5b, which could also be a result of the high iron concentration in the large intestine of CRC patients.

There are many studies supporting that oxidative stress can cause carcinogenesis including CRC^36^. Studies have shown that the oxidative stress in CRC cells is normally higher than that in normal cells^37,38^. According to the GO annotation results, 42 of the 341 differential proteins are related to oxidative stress (Supplementary Table 3). Nicotinamide adenine dinucleotide (NADH) oxidase family has been reported as the major sources of reactive oxygen species (ROS) and reactive nitrogen species (RNS)^39^. Our results show that NADH:flavin oxidoreductases/NADH oxidases from both *Roseburia intestinalis* and *Lachnospira pectinoschiza* were less abundant in the CRC patients compared to healthy crowds (FC(P/H) = 0.31, *P* value = 0.038 and FC(P/H) = 0.089, *P* value = 0.027, respectively) (Fig. 5c), which could be a result of the high concentrations of external ROS and RNS in the large intestine of the CRC patients. Superoxide dismutases (SODs) are important in oxidative stress modulation and act as superoxide scavengers^40^. We found that SODs from *Bacteroides* were more abundant in the CRC patients compared to the healthy crowds (FC(P/H) = 6.06, *P* value = 0.019), Fig. 5d, which could also be a result of the high local oxidative stress in the large intestine of the CRC patients.

## Discussion

Studies have shown that the microbial composition in CRC patients is different from that in healthy crowds^8,9,23^. However, the function of microbes in CRC patients remains poorly understood. Quantitative metaproteomics has been emerging as a powerful approach to characterize microbial function in diseases pathogenesis^41^. Nevertheless, the quantitative metaproteomic characterization of gut microbiome is particularly difficult because of the ultrahigh complexity of the samples. In our experiment, we used a label-free DIA method to identify and quantify gut microbial proteins from fecal samples.

The HMP stool database was used for data analysis, in which the protein entries were translated from gut microbial genes confirmed in healthy crowds (80 males and 59 females). Since no metagenomic sequencing was performed in the work, proteins from species specific to CRC and proteins expressed by microbial genes with mutations specific to CRC may not be identified. As an attempt to identify proteins not included in the HMP database, de novo sequencing was performed to assist database searching against the NCBI nr bacteria database. The SPIDER algorithm in PEAKS is also used to assign the de novo only results to proteins from the HMP, SwissProt human, or NCBI nr bacteria database. The proteins identified from the NCBI nr database (11,994 entries) were used as a complement to the HMP database to ensure that database searching was performed with an optimal protein sequence database for library generation. Since DDA was made by pooling, it might deplete low abundant peptides present in small fraction of samples. Therefore, we also used spectrum-centric approaches to analysis the DIA data to identify peptides that were present in individual samples. The numbers of identifications using spectrum-centric approaches were less than those from the pooled DDA at both the protein group and peptide levels. It has been reported that peptide-centric approaches perform better to exploit highly comprehensive DIA data than spectrum-centric methods^42^. In this metaproteomic study, complex microbiome samples could lead to limited extraction accuracy of precursor-product groups during the spectrum-centric analysis.

The DDA search results and the spectrum-centric DIA results were merged to generate a spectral library for peptide-centric DIA analysis to identify and quantify proteins from each fecal sample in a high throughput manner. Compared to isobaric labeling-based quantitative proteomics, e.g., tandem mass tags (TMT)^43^, DIA is more efficient and provides higher throughput for analysis of large cohorts of samples. TMT allows simultaneous analysis of up to 16 samples. Nevertheless, for experiments with >16 samples (e.g., 28 samples in this study), multiple blocks are required with a reference channel in each block, which can lead to missing values across blocks^44^. In addition, prefractionation is performed on each sample in TMT workflow but only on the pooled sample in DIA workflow for library generation. Compared to label-free quantification based on MS1 peak intensities or spectra count, DIA can provide more reliable quantitative results and wider dynamic ranges^45^. We have identified and quantified in total 30,062 proteins from the intestinal microbes present in the healthy crowds or CRC patients using the DIA proteomic strategy, which is more than the most recently published metaproteomic results on fecal samples. Functional variations of the gut microbiome of the CRC patients compared to the healthy crowds were confirmed by the observed quantitative variations of the microbial proteins related to oxidative stress and iron intake/transport.

Red meat consumption has been considered as one of the major factors inducing CRC^46^. Excessive meat consumption increases the concentration of iron in the intestinal lumen of the host. It has been reported that the iron intake in daily diet can affect tumor growth in mice^47^. And iron accumulated in intestinal tumors of CRC patients may result in tumor growth^29^. It has also been reported that unabsorbed iron in the intestinal lumen might cause gut microbial dysbiosis in CRC patients^48^. Iron acquisition is required for the virulence and colonization of many enteric pathogenic bacteria^49^. An increase in colonic iron concentration may be beneficial for the growth of pathogens rather than probiotics^50^. Lee et al. demonstrated that oral iron-replacement therapy obviously affects the diversity and composition of gut microbiome^51^. TonB-dependent receptors are required for the transport of ferri-siderophores in Gram-negative bacteria^52^. We found that the proteins were significantly increased in concentration in the CRC patients, suggesting an increased iron intake by the gut bacteria of the CRC patients.

Iron metabolism is strictly related to the regulation of oxidative stress by Fenton reaction^53^. Oxidative stress is another leading cause in the pathogenesis of CRC^36^. Excessive production of ROS and RNS can result in lipid oxidation, protein oxidation, nitric oxide production, altered enzyme activity, and DNA damage, thereby inducing gene mutations and cell damage^54^. Oxidative stress is also a key factor in aggravating intestinal dysbiosis^55^. Li et al. demonstrated that fecal microbiome transplantation could eliminate oxidative stress in mice with experimental necrotizing enterocolitis^56^. According to the metaproteomic results of the CRC patients, the concentrations of SOD were significantly increased, which could be a result of high oxidative stress in the intestinal environment. The high oxidative stress can accelerate the progression of CRC. Based on our metaproteomic research, we suggest that not only intestinal cells but also gut bacteria participate in the pathogenesis of CRC through iron intake and oxidative stress regulation.

**Fig. 6:**
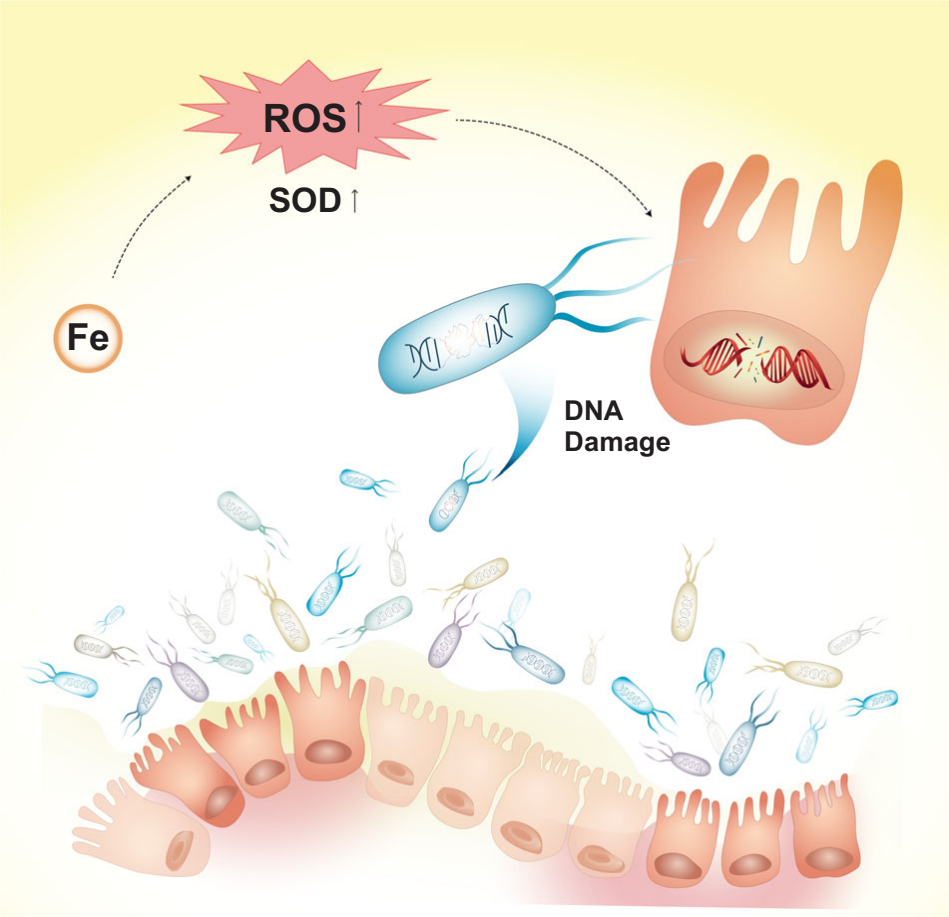
Diagram of gut microbiome in the pathogenesis of CRC. High iron concentration in the intestinal lumen of CRC patients promotes the production of reactive oxygen species (ROS) and results in high oxidative stress. Concentrations of superoxide dismutases (SODs) in the intestinal bacteria are increased. The excessive ROS cause DNA damage of intestinal epithelial cells and probiotic bacteria, which can accelerate the progression of CRC.

Based on previous reports and the metaproteomic study in this work, we propose the global function of gut microbiome in the pathogenesis of CRC as shown in Fig. 6. High iron concentration in the intestinal lumen of CRC patients promotes the colonization of intestinal pathogenic bacteria. Moreover, iron regulates the production of free radicals and involves in oxidative stress regulation through Fenton reaction. With high oxidative stress in intestinal microenvironment, concentrations of antioxidases such as SODs in the intestinal bacteria of CRC patients are increased. The excessive ROS can cause DNA damage of intestinal epithelial cell and probiotic bacteria, which was observed by the increase in concentration of the proteins, e.g., excinuclease UvrABC ATPase subunit and ATP-dependent DNA helicase RecQ, related to DNA damage repair and SOS response in microbes in CRC patients.

In conclusion, we have used quantitative metaproteomics to characterize the gut microbiome of CRC patients and healthy control volunteers. We found 341 microbial proteins of significantly different abundance in CRC patients compared to healthy crowds. Gut microbial proteins related to DNA replication, recombination, and repair were more abundant in CRC patients compared to healthy crowds, which can be associated with high local oxidative stress in the large intestine of CRC patients. Indeed, there were 27 of the 341 differential proteins related to iron intake and transport and 42 related to oxidative stress, and their regulations suggested high local concentration of iron and high oxidative stress. We also suggest some taxonomic abundance variations new to CRC by the quantitative metaproteomics. Our results suggest that gut microbiome can vary in taxonomic abundance and function during the pathogenesis of CRC. Metaproteomics can provide functional information of intestinal microflora and can be a clinical diagnostic method in the future. Clinicians may use the 20 most discriminating proteins to diagnose CRC. Manufacturers can invent a test card for the 20 proteins and patients only need to use their fecal sample to check the risk of CRC in a non-invasive and convenient way. Fecal microbial transplantation to reduce the production of ROS and iron in gut can be a promising method for the prevention of CRC.

## Methods

### Clinical sample collection

Fresh stool samples from newly diagnosed CRC patients prior to any clinical treatments and healthy volunteers were collected in Changhai Hospital (Shanghai, China). All the participants have provided written informed consent prior to the study. Individuals with diabetes, mental disease, gastrointestinal diseases, infections, or who had taken antibiotics during the past 6 weeks before the sample collection were excluded. All participants received only normal Chinese diets 2 months prior to the sample collection. A Chinese diet mainly includes wheat, rice, seasonal vegetables, and lean meats like pork, chicken and beef. Fecal samples were collected in sterile collection tubes in the hospital and sent to our laboratory within 2 h. All fecal samples were stored at −80 °C and handled within 1 month after collection.

### Sample preparation and microbial protein extraction

Microbial cells were enriched from the fecal samples by differential centrifugation according to a method reported by Tanca et al.^57^ Briefly, fecal samples were suspended in 45 mL phosphate-buffered saline (PBS), vortexed for 45 min, and then centrifuged at 500 × *g* for 5 min to remove food debris. The supernatant was collected and stored at 4 °C. The pellets were resuspended in 45 mL PBS, and the above procedure was repeated twice. For each sample, 3 tubes of supernatant were collected, combined, and centrifuged at 10,000 × *g* for 10 min. The pellets were collected and washed by 2 mL deionized water.

The final pellets were frozen in liquid nitrogen and grinded into powder. The powder was collected and suspended in a sodium dodecyl sulfate (SDS)-based buffer (2% SDS, 100 mM dithiothreitol (DTT) in 20 mM tris-HCl, pH = 8.8) at 1:10 (w:v), vortexed, and heated at 95 °C for 30 min. The suspension was centrifuged at 20,000 × *g* for 10 min at 4 °C, and the supernatant was collected for protein extraction. Proteins were extracted by acetone precipitation (6 mL acetone per 1 mL sample) overnight. The purified proteins were collected by centrifugation at 12,000 × *g* for 10 min and redissolved in 7 M Urea and 1% SDS aqueous solution. The final concentrations of the protein samples were adjusted to 1 mg/mL. Bicinchoninic acid (Beyotime, Shanghai) method was used to quantify the extracted proteins, and SDS polyacrylamide gel electrophoresis was used to evaluate the quality of the protein extracts.

The protein extracts were reduced by DTT (5 μL 200 mM DTT per 100 μL sample), alkylated by iodoacetamide (IAA) (20 μL 200 mM IAA per 100 μL sample), and then subjected to trypsin digestion (20 μg trypsin (from Beijing Hualishi Technology Ltd) per 1 mg protein) overnight by following a standard in-solution tryptic digestion protocol^58^. Peptides from each sample were purified and concentrated using Pierce C18 spin columns (Thermo Fisher Scientific, Rockford, USA) and then quantified using Pierce Quantitative Colorimetric Peptide Assay (Thermo Fisher Scientific, Rockford, USA).

### Liquid chromatography (LC)-MS/MS analysis

All samples were analyzed by an online nanospray Orbitrap Fusion Lumos Tribrid mass spectrometer (Thermo Fisher Scientific, MA, USA) coupled with a Nano ACQUITY UPLC system (Waters Corporation, Milford, MA). Ten μg peptides of each sample were analyzed in DIA mode. The peptides were redissolved in 50 μL solvent A (0.1% formic acid in water) spiked with 1× iRT standard (iRT Kit; Biognosys, Schlieren, Switzerland). Ten μL peptide sample was loaded to an Acclaim PepMap C18 column (75 μm × 25 cm) and separated with a 120-min linear gradient, from 3% to 32% solvent B (0.1% formic acid in acetonitrile). The column flow rate was maintained at 300 nL/min and the column temperature was maintained at 40 °C. The electrospray voltage of 2100 V was used. The full scan was performed for *m*/*z* 350–1200 with the resolution of 120,000 at *m*/*z* = 200, and the maximum injection time was 50 ms. The MS/MS scan was performed with higher-energy collision-activated dissociation for *m*/*z* 200–2000 with the resolution of 30,000 at *m*/*z* = 200, and the maximum injection time was 64 ms. The collision energy was 35%, and the stepped collision energy was 5%. DIA was performed with 39 variable isolation windows with 1 Da overlap, and the total cycle time was 3 s.

DDA mode was used for building a spectral library for protein identification and quantification by DIA. Ten μg of peptides from each sample were combined, and the mixture was redissolved in 80 μL buffer C (20 mM ammonium formate in water, pH = 10.0 adjusted by ammonium hydroxide). Then the combined peptide solution was subjected to a high-pH reversed phase LC fractionation using an Ultimate 3000 system (Thermo Fisher Scientific, MA, USA) with an XBridge C18 column (4.6 mm × 250 mm, 5 μm) (Waters Corporation, MA, USA). A linear gradient was used: 5–45% buffer D (20 mM ammonium formate in 80% acetonitrile, pH = 10.0 adjusted by ammonium hydroxide) in 40 min. The column flow rate was maintained at 1 mL/min and the column temperature was maintained at 30 °C. Fractions were continuously collected each 4 min during the gradient. Each fraction was dried in a vacuum-freezing dryer, redissolved in 50 μL solvent A (0.1% formic acid in water), and then subjected to LC-MS/MS analysis. The injection volume was 5 μL. Dynamic exclusion was enabled within 30 s duration. MS/MS scan was performed with 1.6 Da isolation window widths. The other MS parameters as well as LC gradient conditions and LC column were the same as those in DIA experiments.

### LC-MS/MS data analysis

DDA data were first analyzed using PEAKS Studio (version X, Bioinformatics Solutions Inc., Canada). MS1 tolerance was set to 7 ppm, and MS/MS tolerance was 0.02 Da. All the DDA MS/MS spectra were searched against the database of stool microbial proteomes downloaded from HMP (4,854,034 protein entries, https://hmpdacc.org/, access data November 2017). Those identified with PSM-level false discovery rate (FDR) <1% were assigned to a peptide from the HMP database. All the other DDA MS/MS spectra with de novo sequencing average local confidence (ALC) threshold >80% were then searched against the database combining SwissProt human (20,416 protein entries, https://www.uniprot.org/, access date July 2019) and the NCBI nr bacteria (78,778,748 protein entries, https://www.ncbi.nlm.nih.gov/, access date July 2019). Those identified with PSM-level FDR <1% were assigned to a peptide from the SwissProt human plus NCBI nr bacteria database. The remaining spectra with de novo sequencing ALC threshold >80% were reported as de novo only results. Some of the de novo only results were assigned to proteins from the HMP, SwissProt human, or NCBI nr bacteria database using the SPIDER algorithm in PEAKS. The rests were reported as not assigned as shown in Supplementary Fig. 1.

Then the DDA and DIA raw data were processed and analyzed by SpectroMine (version 1.0.21621, Biognosys AG, Switzerland) with default settings to search against a database combined from the HMP and the identified NCBI nr proteins (11,994 entries). Trypsin was the digestion enzyme. Carbamidomethyl (C) was specified as fixed modification. Oxidation (M) was specified as variable modification. FDR cut-off on PSM, peptide, and protein group level were 1%. The DDA and DIA spectrum-centric search results were combined to generate a spectral library for peptide-centric DIA analysis.

Raw data of DIA were then processed and analyzed by Spectronaut (version 13.10.191212, Biognosys AG, Switzerland) with default settings. RT prediction type was set to dynamic iRT. Decoy generation was set to mutated. Interference correction on MS2 level was enabled. The top 3 filtered peptides that passed the 1% *Q*-value cut-off were used to calculate the major group quantities. Protein inference was performed with IDPicker algorithm^59^ implemented in Spectronaut. Only the leading protein (with the strongest evidence and ranked first by Spectronaut) in each protein group was taken into consideration in subsequent statistical, functional, and taxonomic analysis. Quality control was performed with QuiC (Biognosys AG, Switzerland).

### Statistical, functional, and taxonomic analysis

Statistical analysis was performed using MetaboAnalyst (version 4.0, https://www.metaboanalyst.ca/)^60^. ROC curves were generated by 100 rounds of Monte-Carlo cross-validation using balanced sub-sampling. In each round, 2/3 of the samples were used to build classification models, which was validated on the 1/3 of the samples that were left out. LSVM was used as a classification method. Annotation of the differentially expressed proteins was performed with Blast2GO integrated in OmicsBox (version 1.2, https://www.biobam.com/omicsbox/)^33^. The top hits were assigned to the query proteins. In addition, eggNOG (version 4.5.1, http://eggnogdb.embl.de/)^28^ was used to perform COG annotation. Identified peptides were subjected to Unipept (version 4.0, https://unipept.ugent.be/) for taxonomic analysis using the lowest common ancestor approach^61^. Abundance of each taxon was determined by summing the intensities of all peptides corresponding to the taxon. Data visualization was conducted with R (version 3.5.1, https://www.r-project.org/), ggplot2 (https://github.com/tidyverse/ggplot2), and AntV G2 (https://github.com/antvis/g2).

### Ethical statement

The subjects gave their informed consent for using the biological material for research purposes. The study protocol was approved by the Shanghai Changhai Hospital Ethics Committee.

### Reporting summary

Further information on research design is available in the Nature Research Reporting Summary linked to this article.

## Supplementary information

Supplementary Information

Supplementary Data 1

Supplementary Data 2

Supplementary Data 3

Supplementary Data 4

Supplementary Data 5

Supplementary Data 6

## Data Availability

The raw mass spectrometric data, the spectral libraries for DIA proteomic analysis, the saved projects from Spectronaut, and the FASTA files including all protein sequence entries used in this study from Human Microbiome Project have been deposited to the ProteomeXchange Consortium (http://www.proteomexchange.org/) via the iProX^62^ partner repository with the data set identifier PXD013386/IPX0001564000. The source data underlying Figs 1e, 2, 3a, 4, and 5 are provided as a Source Data file.

## Source Data

Source Data
Reporting Summary
