# Supplementary material for: Metaproteomics characterizes human gut microbiome function in colorectal cancer
Source: NPJ Biofilms Microbiomes. 2020 Mar 24;6:14. doi: 10.1038/s41522-020-0123-4 (PMC7093434; doi:10.1038/s41522-020-0123-4)
Supplement: Supplementary file 1 — Supplementary Information [file 41522_2020_123_MOESM1_ESM.pdf]

Supplementary information for

Metaproteomics Characterizes Human Gut Microbiome Function in Colorectal Cancer

Shuping Long [1,2,3#], Yi Yang [1,#], Chengpin Shen [4], Yiwen Wang [1], Anmei Deng [2], Qin Qin [2,*] and Liang Qiao [1,*]

1. Department of Chemistry, Shanghai Stomatological Hospital, Fudan University, Shanghai, China

2. Changhai Hospital, The Naval Military Medical University, Shanghai, China;

3. Department of Clinical Laboratory Medicine, Shanghai Tenth People's Hospital of Tongji University, Shanghai, China

4. Shanghai Omicsolution Co., Ltd., Shanghai, China

[*]Address correspondence to these authors at: liang_qiao@fudan.edu.cn for Dr. Liang Qiao or qinq78@163.com for Dr. Qin Qin.

[#]These authors contribute equally to the work.

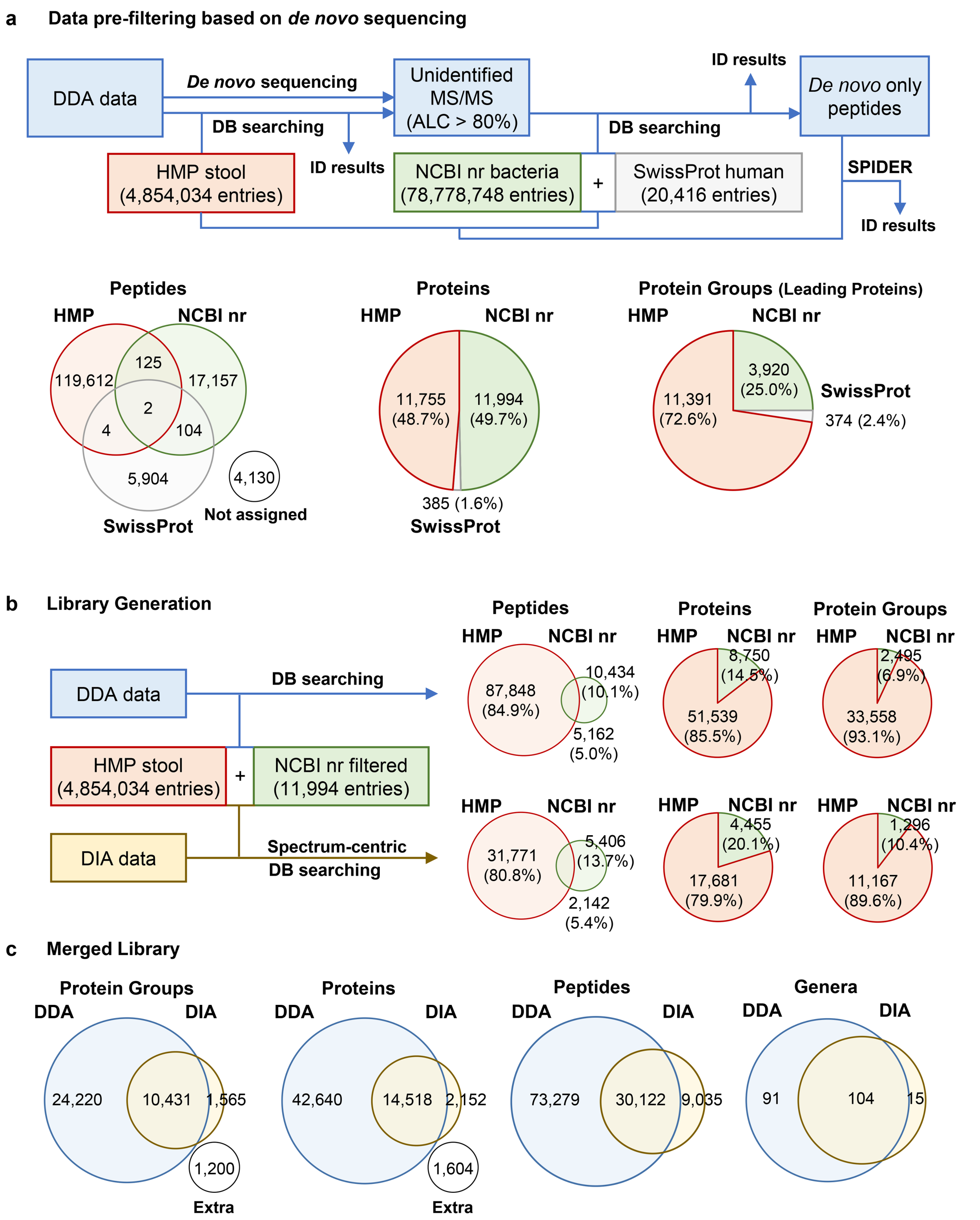

Supplementary Fig. 1. Workflow of spectral library generation. (**a**) Results of *de novo* sequencing assisted database searching by PEAKS on the pooled DDA data against successively the database of stool from Human Microbiome Project (HMP) and the database combining the NCBI nr bacteria plus the SwissProt human. SPIDER matching was applied on the *de novo* only results. (**b**) Results of DDA database searching and DIA spectrum-centric analysis by SpectroMine against a database combining the HMP and the identified NCBI nr proteins in **a** (11,994 entries). (**c**) The number of proteins (groups), peptides, and genera in the library merged from the DDA and DIA database searching results in **b**. Spectronaut applied filters to the search

results when generating spectral libraries. As protein inference was re-performed, the merged library contained proteins/protein groups not included in the previous DDA and DIA database searching results.

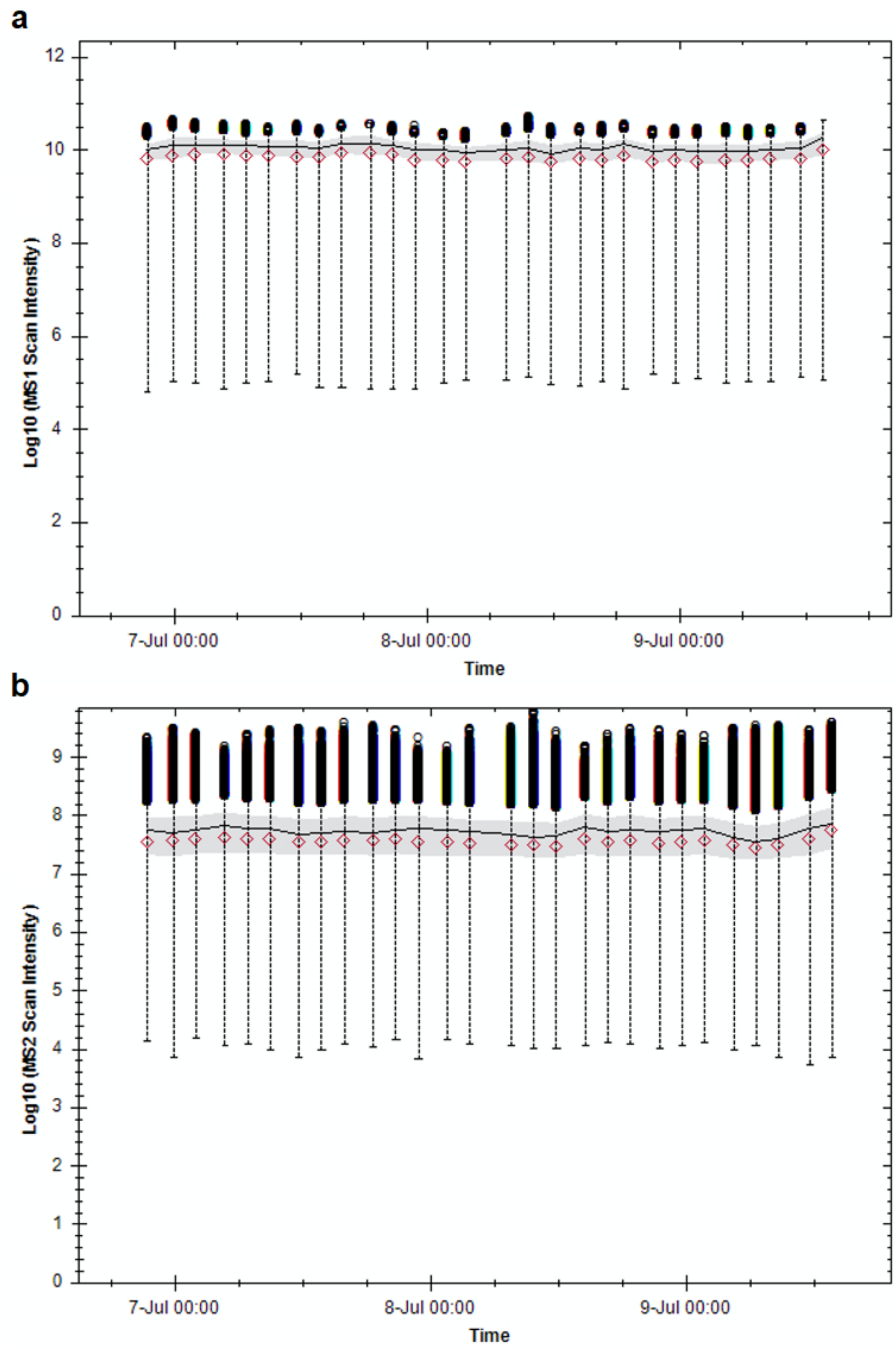

Supplementary Fig. 2. Quality control of LC-MS performance using iRT peptides. (**a**) Sum total of intensities per MS1 scan. (**b**) Sum total of intensities per MS2 scan. The black solid line represents median values, the grey belt represents quantiles (*Q*1 and *Q*3), the whiskers extend from the *Q*1 to *Q*1−1.5 *IQR* and from *Q*3 to *Q*3+1.5 *IQR* (where *IQR* is the inter-quartile range), the circles represent outliers, and the red diamonds represent mean values.

Supplementary Table 1. Cohort characters.

	Healthy control	CRC
Age (mean ± sd)	56.90 ± 14.98	59.64 ± 9.81
Body weight (mean ± sd)	69.41 ± 9.71	68.93 ± 8.39

Supplementary Table 2. Differential proteins between CRC patients group (P) and healthy controls group (H) related to iron intake and transport.

Accession	Description	Organism	*FC*(P/H)	*P*
SRS011239.197003-T1-C	Bacterioferritin	Parabacteroides	276	0.049
SRS015663.106262-T1-C	FprA family A-type flavoprotein	Subdoligranulum sp.	186	0.013
SRS024331.38208-T1-C	hemerythrin domain-containing protein	Bacteroides	4.71	0.044
SRS019601.19650-T1-C	SusC/RagA family TonB-linked outer membrane protein	Bacteroides vulgatus	3.57	0.044
SRS016056.14438-T1-C	SusC/RagA family TonB-linked outer membrane protein	Bacteroides	3.47	0.015
SRS015578.78981-T1-C	TonB-dependent receptor	Bacteroides	2.35	0.029
SRS015217.85864-T1-C	fumarate hydratase	Bacteroides	2.33	0.008
SRS019601.63321-T1-C	NifU-related domain containing protein	Firmicutes	0.453	0.028
SRS015663.49187-T1-C	hemerythrin	Ruminococcus	0.436	0.046
SRS047014.124405-T1-C	iron-containing alcohol dehydrogenase	Faecalibacterium prausnitzii	0.411	0.038
SRS012273.61913-T1-C	3-isopropylmalate dehydratase large subunit	Blautia obeum	0.400	0.031
SRS019787.19815-T1-C	4Fe-4S dicluster domain-containing protein	Faecalibacterium prausnitzii	0.355	0.037
SRS019397.44624-T1-C	pyruvate:ferredoxin (flavodoxin) oxidoreductase	Clostridium	0.272	0.038
SRS014235.36887-T1-C	TonB-dependent receptor	Alistipes putredinis	0.249	0.036
SRS015190.45280-T1-C	ferritin	Bacteroides	0.239	0.046
SRS021948.238782-T1-C	SusC/RagA family TonB-linked outer membrane protein	Parabacteroides	0.145	0.042
SRS017433.34051-T1-C	pyruvate ferredoxin oxidoreductase	Fusobacterium mortiferum	0.144	0.032
SRS020233.345763-T1-C	2-oxoglutarate ferredoxin oxidoreductase subunit beta	Phascolarctobacterium sp.	0.140	0.041
SRS015264.48753-T1-C	pyruvate:ferredoxin (flavodoxin) oxidoreductase	Faecalibacterium sp.	0.119	0.030
SRS015794.38648-T1-C	rubrerythrin family protein	Alistipes putredinis	0.096	0.045
SRS020328.18596-T1-C	pyruvate:ferredoxin (flavodoxin) oxidoreductase	Ruminococcaceae bacterium	0.095	0.012
SRS051031.43732-T1-C	NADH flavin oxidoreductase NADH oxidase	Lachnospira pectinoschiza	0.089	0.027
SRS023914.10992-T1-C	TonB-dependent receptor	Prevotella copri	0.039	0.037
SRS018351.56581-T1-C	Fe3+-hydroxamate ABC transporter substrate-binding protein	Ruminococcus	0.031	0.018
SRS014459.187501-T1-C	(Fe-S)-binding protein	Bilophila sp.	0.009	0.024

SRS014923.30252-T1-C	FeS cluster assembly scaffold protein NifU	Dorea	0.007	0.043
CDA55034.1	TonB-linked outer membrane protein SusC/RagA family	Prevotella sp.	0.128	0.031

Supplementary Table 3. Differential proteins between CRC patients group (P) and healthy control group (H) related to oxidative stress.

Accession	Description	Organism	*FC*(P/H)	*P*
SRS024435.62429-T1-C	citrate/2-methylcitrate synthase	Bacteroides dorei	174	0.021
SRS023526.15548-T1-C	shikimate dehydrogenase	Prevotella sp.	168	0.041
SRS020869.130274-T1-C	L-arabinose isomerase	Bifidobacterium longum	25.7	0.049
SRS020233.228920-T1-C	NADP-specific glutamate dehydrogenase	unclassified Muribaculaceae	11.7	0.004
SRS015217.46779-T1-C	Gfo/Idh/MocA family oxidoreductase	Oscillospiraceae	8.98	0.038
SRS054956.79161-T1-C	beta-aspartyl-peptidase	Coprococcus comes	7.31	0.040
SRS024132.15018-T1-C	superoxide dismutase	Bacteroides	6.06	0.019
SRS015782.46050-T1-C	aspartate-semialdehyde dehydrogenase	Bacteroides plebeius	4.76	0.042
SRS015217.220697-T1-C	3-hydroxybutyryl-CoA dehydrogenase	Oscillospiraceae	4.74	0.045
SRS019582.96270-T1-C	glycosyl hydrolase family 109 protein 1	Bacteroides	4.70	0.021
SRS018817.12115-T1-C	bifunctional methylenetetrahydrofolate dehydrogenase/methenyltetrahydrofolate cyclohydrolase FolD	Bacteroides	4.65	0.044
SRS011586.20489-T1-C	flavodoxin FldA	Bacteroides	3.90	0.048
SRS015264.66232-T1-C	aspartate-semialdehyde dehydrogenase	Bacteroides	3.53	0.040
SRS014613.14763-T1-C	4-hydroxy-tetrahydrodipicolinate reductase	Bacteroides vulgatus	2.67	0.047
SRS015217.49952-T1-C	4-hydroxythreonine-4-phosphate dehydrogenase PdxA	Faecalibacterium prausnitzii	0.498	0.021
SRS014923.48001-T1-C	NADH flavin oxidoreductase NADH oxidase	Roseburia intestinalis	0.310	0.038
SRS064276.22511-T1-C	alpha-hydroxy-acid oxidizing protein	Faecalibacterium prausnitzii	0.282	0.042
SRS019397.44624-T1-C	pyruvate:ferredoxin (flavodoxin) oxidoreductase	Clostridium	0.272	0.038
SRS055982.21013-T1-C	2-hydroxy-3-oxopropionate reductase	Faecalibacterium sp.	0.215	0.032
SRS013158.16570-T1-C	RnfABCDGE type electron transport complex subunit D	Bacteroides stercoris	0.214	0.014
SRS014313.5645-T1-C	fumarate reductase/succinate dehydrogenase flavoprotein subunit	Bacteroides caccae	0.167	0.015
SRS015065.63907-T1-C	gluconate 5-dehydrogenase	Bacteroides coprocola	0.156	0.047
SRS011134.88333-T1-C	D-2-hydroxyacid dehydrogenase	Dorea longicatena	0.153	0.018
SRS017433.34051-T1-C	pyruvate ferredoxin oxidoreductase	Fusobacterium mortiferum	0.144	0.032
SRS020233.345763-T1-C	2-oxoglutarate ferredoxin oxidoreductase subunit beta	Phascolarctobacterium sp.	0.140	0.041
SRS015264.48753-T1-C	pyruvate:ferredoxin (flavodoxin) oxidoreductase	Faecalibacterium sp.	0.119	0.030

SRS015794.38648-T1-C	rubrerythrin family protein	Alistipes putredinis	0.096	0.045
SRS020328.18596-T1-C	pyruvate:ferredoxin (flavodoxin) oxidoreductase	Ruminococcaceae bacterium	0.095	0.012
SRS017191.53173-T1-C	gluconate 5-dehydrogenase	Roseburia sp.	0.091	0.023
SRS051031.43732-T1-C	NADH flavin oxidoreductase NADH oxidase	Lachnospira pectinoschiza	0.089	0.027
SRS016056.25975-T1-C	aminomethyl-transferring glycine dehydrogenase	Alistipes putredinis	0.078	0.021
SRS012902.35783-T1-C	ketol-acid reductoisomerase	Parasutterella excrementihominis	0.072	0.036
SRS024435.1474-T1-C	NADH peroxidase	Firmicutes	0.048	0.029
SRS015663.36908-T1-C	3-hydroxybutyryl-CoA dehydrogenase	Eubacterium ventriosum	0.033	0.015
SRS017521.216871-T1-C	aminomethyl-transferring glycine dehydrogenase subunit GcvPA	Clostridium sp.	0.017	0.034
SRS042284.10234-T1-C	Nitronate monooxygenase	Bacteroides	0.011	0.045
SRS050422.126124-T1-C	AhpC/TSA family protein	Alistipes putredinis	0.009	0.033
SRS015578.72475-T1-C	IMP dehydrogenase	Ruminococcus bicirculans	0.005	0.050
SRS023526.167479-T1-C	NAD(P)H-dependent oxidoreductase subunit E	Clostridiales	0.005	0.014
SRS015578.60198-T1-C	FAD-dependent oxidoreductase	Ruminococcus sp.	0.004	0.047
SRS015663.21622-T1-C	SusC/RagA family TonB-linked outer membrane protein	Bacteroides sp.	0.001	0.043
OKY96878.1	superoxide dismutase	Alistipes putredinis	0.133	0.046

Captions for the Supplementary Data Sets and the Source Data file

File Name: Supplementary Data 1
Description: Identification results of *de novo* sequencing assisted database searching on the pooled DDA data.

File Name: Supplementary Data 2.
Description: DDA and spectrum-centric DIA search results used for library generation.

File Name: Supplementary Data 3.
Description: Peptide-centric DIA analysis results.

File Name: Supplementary Data 4.
Description: Genera of microbes identified in the healthy crowds and CRC patients.

File Name: Supplementary Data 5.
Description: Differential proteins between the CRC patients and healthy crowds.

File Name: Supplementary Data 6.
Description: Information on the phylum, class, order, and family of the labeled numbers in Figure 2.

File Name: Source Data.
Description: The source data underlying Fig. 1e, 2, 3a, 4 and 5.
